# Optimization and Modeling of Helium Recovery from Natural Gas Through Hydrate-Based Gas Separation

**DOI:** 10.3390/molecules31091486

**Published:** 2026-04-29

**Authors:** Yiwei Wang, Lina Meng, Zheng Liu, Shiguang Fan, Jinqiang Liang, Zhen Xu, Qiang Sun, Xuqiang Guo

**Affiliations:** 1 State Key Laboratory of Heavy Oil Processing, China University of Petroleum Beijing at Karamay, Karamay 834000, China; wyw@cup.edu.cn (Y.W.); 18082848252@163.com (L.M.); sgf@cupk.edu.cn (S.F.); jqliang2024@cupk.edu.cn (J.L.); guoxq@cup.edu.cn (X.G.); 2 State Key Laboratory of Heavy Oil Processing, China University of Petroleum (Beijing), Beijing 102249, China; lz13071051028@163.com (Z.L.); sunq@cup.edu.cn (Q.S.); 3 College of Mechanical and Transportation Engineering, China University of Petroleum (Beijing), Beijing 102249, China

**Keywords:** hydrate-based gas separation, helium recovery, natural gas, mathematical model

## Abstract

As a finite strategic resource, helium is extracted from natural gas (NG). The concentration of helium in NG is very low, which makes helium hard to separate. The hydrate-based gas separation (HBGS) was proposed as a promising method for the separation of the NG with low helium content in this work. This work systematically investigated the HBGS of helium from simulated NG. The thermodynamic analysis reveals that the existence of 5.00 mol% tetrahydrofuran (THF) in the liquid phase decreased the gas–liquid–hydrate equilibrium pressure by 92.11%, compared to the deionized water system. The single-stage HBGS experimental results show that high THF concentration, low temperature, and high pressure benefited the gas processing capacity and helium purification, but they led to a low helium recovery rate. The best HBGS performance was limited by the “hydrate shell effect”. The decrease in gas–liquid ratio led to an increase in helium concentration without losing the gas processing capacity, but it caused a decrease in the helium recovery rate. Through three-stage HBGS optimization, the helium concentration was increased from 0.54 mol% to 13.54 mol% (a 25.07-fold enrichment), and a total helium recovery of 87.34% was achieved. The mathematical model proposed in this work accurately predicts the performance of HGBS with 2.09% average relative error compared to the experimental data.

## 1. Introduction

As a strategically critical resource with limited global availability, helium (He) enables the operations in aerospace, defense infrastructure, and precision industrial applications [1,2,3]. Current estimates indicate the global helium resources with an approximate total of 39.79 billion cubic meters [4,5]. Natural gas (NG) reservoirs represent the primary viable source, typically containing helium between 0.3% and 1.5% [4]. Economic extraction requires a helium concentration higher than 0.3%, making helium-containing NG essential for a sustainable global helium supply [6].

In the helium-producing countries with significant liquefied natural gas (LNG) exports (e.g., the United States), helium is recovered as a by-product during cryogenic distillation of NG to LNG [7,8,9]. The helium-rich off-gas (1 mol%~3 mol% helium) of LNG undergoes pre-concentration to 50 mol%~80 mol% helium via cryogenic methods, followed by adsorption purification to 99.995 mol% purity [10]. However, that energy-intensive phase-change process is economically unviable for countries without large-scale LNG infrastructure, such as China. In the countries that recover helium directly from NG, cryogenic distillation is used to enrich the helium concentration from less than 1% to higher than 10% to produce crude helium gas. Then, the crude helium gas can be efficiently purified by pressure swing adsorption (PSA) and membrane to produce high-purity helium gas [11,12,13,14,15,16]. Though cryogenic distillation is energy-intensive, it is still chosen as the method for crude helium production, because the low helium content and the acid components make NG unsuitable to be separated directly by PSA and membrane [17,18]. According to the selectivity of the hydrate cage for the gas molecules of different sizes, researchers have confirmed that hydrate-based gas separation (HBGS) is economically feasible for the crude industrial gas production [19,20]. HBGS may offer a new economical route for crude helium production [21,22].

Hydrates constitute variable–composition compounds characterized by a crystalline clathrate structure [23,24,25]. HBGS uses water as the working medium and is operated under mild conditions [26,27]. Chen et al. systematically investigated the separation of carbon dioxide and hydrogen, and the hydrogen concentration was increased from 40 mol% to 57.31% with a separation efficiency of 42.26% [28]. Duan et al. proposed a metal–organic framework (MOF) system that provides important insights for advancing efficient NG capture by hydrate [29]. To reduce the energy consumption of HBGS, tetrahydrofuran (THF) and tetrabutylammonium bromide (TBAB) were used to promote the hydrate formation [30,31]. Additionally, mathematical models of HBGS have been developed to predict gas separation performance by researchers [32,33]. Previous investigations have established that gas separation via HBGS is a promising technology.

**Figure 1:**
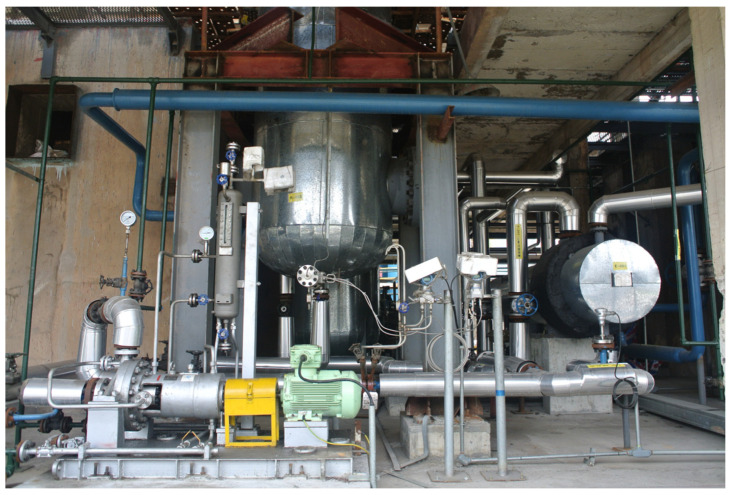
On-site device at Sinopec Maoming Petrochemical Company.

Our team set up an industrial HBGS side-line for the Sinopec Maoming Petrochemical Company, as shown in Figure 1. That demonstration illustrates the simultaneous capture of light hydrocarbons and enrichment of hydrogen from diesel hydrogenation tail gas using THF as promoter. Due to the similar properties of hydrogen and helium, the successful operation of this industrial HBGS side-line has provided an innovative approach for extracting helium from natural gas using the hydrate method.

The hydrate-based separation method has significant potential in the separation of low helium content gas; however, it is not highly efficient for the separation of high helium content gas, because the high helium content makes hydrate hard to form [18]. PSA is effective at the separation of high helium content gas, but it is not highly efficient for the separation of the gas mixture in which the helium content is only 0.5 mol%, because PSA purifies helium through the adsorption of the components other than helium, whereas the adsorption capacity of the adsorbent is limited [17]. For those reasons, the hydrate-based septation method can be used to produce the feed gas (crude helium) for PSA from low helium content gas. This work focuses on the production of crude helium gas from a simulated NG (97.89 mol% CH_4_, 1.65 mol% C_2_H_6_, 0.54 mol% He) using the HBGS method. The gas–liquid–hydrate equilibrium (GLHE) conditions for the simulated NG were determined across varying THF concentrations to assess pressure reduction. According to the literature [17], PSA is highly efficient when the helium concentration in its feed gas is higher than 10.00 mol%, so that 10.00 mol% was determined as the target of the hydrate-based separation in this work. Therefore, the effectiveness of increasing the helium concentration from 0.54 mol% to 10.00 mol% was verified using the three-stage HBGS method. A mathematical model was developed to predict the performance of helium extraction from NG and to guide the design of the process and equipment.

## 2. Results

### 2.1. The GLHE Conditions of Helium-Containing NG

**Figure 2:**
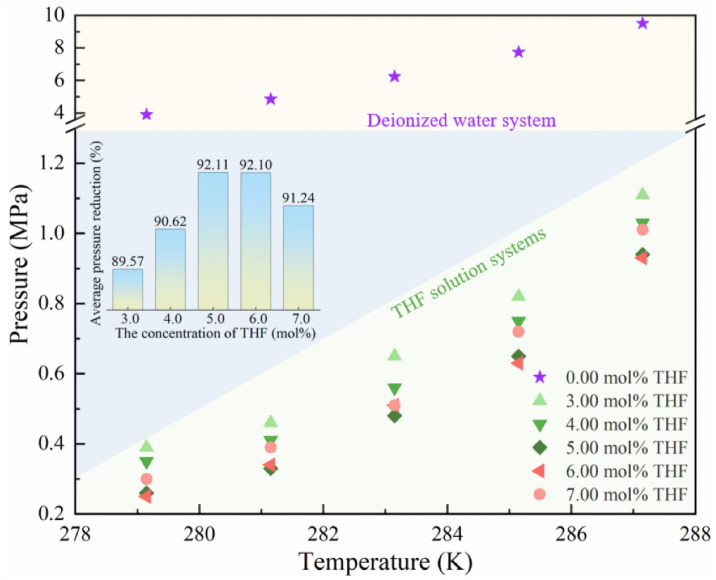
The GLHE condition of helium-containing NG with the solutions with different THF concentrations.

The precise determination of the GLHE conditions for helium-containing NG is critical for optimizing HBGS process parameters. Figure 2 delineates the experimentally measured GLHE pressure for a simulated NG across varying THF concentrations. A salient observation is the substantially higher hydrate formation pressure required in deionized water systems compared to THF-containing systems at equivalent temperatures. The average GLHE pressure for helium-containing NG hydrates formed in deionized water was measured 6.45 MPa over the temperature range 279.15 K~287.15 K. This necessitates either significantly depressed operating temperature or elevated pressure for effective gas separation using deionized water, inevitably leading to increased energy consumption on compression and refrigeration infrastructure. Conversely, the introduction of THF had a pronounced thermodynamic promotion effect, markedly decreasing GLHE pressures. This was directly attributable to THF’s well-established role as a thermodynamic hydrate promoter [34].

In addition, the GLHE pressure of helium-containing NG hydrate varies with THF concentration. The average pressure reduction (*APR*) is used to characterize the decreasing range of the GLHE pressure with different THF concentrations, which is calculated as follows:

(1) $$APR=\frac{1}{N}\sum_{1}^{N}\frac{P_{THF}-P_{H_{2}O}}{P_{H_{2}O}}$$

where *N* indicates the number of data points. $P_{THF}$ and $P_{H_{2}O}$ represent GLHE pressures in the THF solution system and the deionized water system at the same temperature, respectively. *APR* increases from 89.57% (3.00 mol% THF) to 92.11% (5.00 mol% THF), indicating that the GLHE pressure decreases. The reason is that THF molecules, being soluble in water and of appropriate molecular size, readily incorporate into the large cavities of the hydrate lattice formed by NG components. That occupancy stabilizes the hydrate framework at significantly lower pressure and higher temperatures than would be feasible in deionized water. *APR* decreases from 92.11% (5.00 mol% THF) to 91.24% (7.00 mol% THF), indicating that the GLHE pressure increases. The reason is that substantial THF molecules in the liquid phase compromise the integrity of the water molecular network through THF–water hydrogen bonding, and that depresses water activity and elevates GLHE pressure.

To decrease the GLHE pressure, the best THF concentration is 5.00 mol%; the GLHE pressure of the three-component simulated NG ranges from 0.26 MPa to 0.94 MPa, and the corresponding temperature ranges from 279.15 K to 287.15 K in a 5.00 mol% THF solution. That makes the hydrate formation conditions mild, which is conducive to decreasing energy consumption and equipment costs.

### 2.2. The Performance of Single-Stage HBGS for the Helium Extraction from NG

#### 2.2.1. The Performance of HBGS at Different THF Concentrations

THF effectively decreases the GLHE pressure of helium-containing NG. The performance of HBGS at different THF concentrations on the helium extraction still needs to be investigated. The separation performances were investigated over THF concentrations of 3.50 mol% to 5.50 mol% THF solutions. The selected range of THF concentration covered the THF concentration for the lowest GLHE pressure. Experimental conditions comprised a gas–liquid ratio (*GLR*) of 110 Nm^3^/m^3^, with system temperature and pressure maintained at 281.15 K and 3.00 MPa. The helium separation results at different THF concentrations are summarized in Table 1.

Table 1 shows that the amount of gas captured (*GSC*) is positively correlated with THF concentration. The *GSC* increases from 53.75 Nm^3^/m^3^ at 3.50 mol% THF to 75.65 N m^3^/m^3^ at 5.00 mol% THF, and the incremental gradient is about 14.6 (Nm^3^/m^3^)∙mol^−1^. The reason is that increased THF concentrations enhance the thermodynamic driving force for hydrate formation, concurrently increasing hydrate mole production. However, the *GSC* only increases from 75.65 Nm^3^/m^3^ at 5.00 mol% THF to 76.86 Nm^3^/m^3^ at 5.50 mol% THF, and the incremental gradient is only about 2.42 (Nm^3^/m^3^)∙mol^−1^. This is because the driving force of hydrate formation is too high, and hydrate forms a “hydrate shell”, which hinders the efficiency of gas–liquid mass transfer [35,36]. In addition, at high THF concentration, the THF hydrate cage crystals remain stable under the condition of no gas molecule adsorption, increasing the amount of the empty hydrate cavities; thus, less gas is captured per unit hydrate.

With the increase in *GSC*, CH_4_ and C_2_H_6_ molecules are captured in the hydrates, while helium molecules are enriched in the equilibrium gas. Therefore, the helium concentration in equilibrium gas increases from 1.04% at 3.50 mol% THF to 1.66% at 5.00 mol% THF, and the recovery factor of helium (*RF*) of helium decreases from 98.49% to 96.00%. However, due to the entrainment of helium molecules into the hydrate slurries, the helium concentration decreases from 1.66 mol% at 5.00 mol% THF to 1.65 mol% at 5.50 mol% THF, and the *RF* of helium decreases significantly to 92.06%.

The gas mixture of CH_4_, C_2_H_6_, and helium released from the hydrate after hydrate dissociation is called dissociated gas. The helium concentration in dissociated gas is about 0.03 mol% throughout 3.50 mol% to 5.00 mol% THF solutions, which shows no significant change. However, the helium concentration increased by 2-fold (from 0.03 mol% to 0.06 mol%) with increasing THF from 5.00 mol% to 5.50 mol%. This originates from hydrate shell obstruction of gas migration in high-THF systems, consequently augmenting entrapped helium in slurry phases.

**Figure 3:**
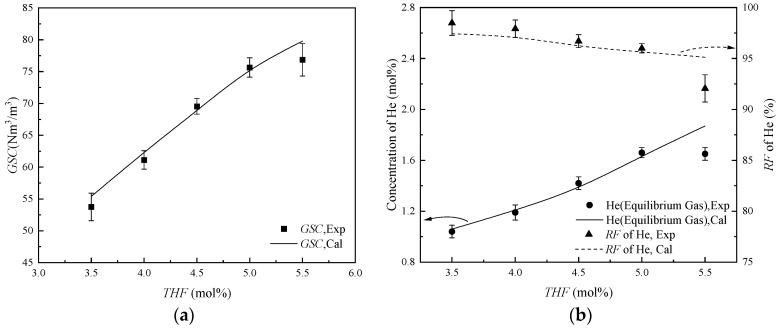
The helium separation performance from NG at different THF concentrations. (**a**) The values of *GSC*; (**b**) The values of concentration of He and *RF* of He.

The comparison between the model-predicted and the experiment-measured on the indexes of *GSC*, helium concentration, and *RF* of helium is shown in Figure 3. The maximum relative error (*MRE*) is 13.33% for the 5.50 mol% THF system, which may be because the hydration shell effect made many small bubbles in the liquid phase covered by hydrate, so that the gas in the bubbles was trapped in the hydrate slurry phase and did not return to the bulk gas phase, which increased the number of helium molecules in the hydrated slurry. With an average relative error (*ARE*) of 2.49%, the mathematical model demonstrates a solid predictive accuracy for helium separation performance from NG at varying THF concentrations. Considering the industrial application of using the HBGS method to separate helium from helium-containing NG, it is required that the hydrate has a high *GSC* while satisfying the improvement of concentration and *RF* of helium. A higher *GSC* is conducive to increasing the gas processing capacity, but is not conducive to improving the *RF* of helium. Considering the scarcity of helium resources, the helium concentration should be increased as much as possible on the assumption that the decrease in the *RF* of helium is not large. Therefore, a 5.00 mol% THF system was selected as the concentration for subsequent experiments.

#### 2.2.2. The Performance of HBGS at Different GLR

*GLR* serves as a key process parameter for quantifying NG processing capacity on the helium extraction from NG by HBGS. The separation performances were investigated in the *GLR* range of 100 Nm^3^/m^3^ to 140 Nm^3^/m^3^. The THF concentration, temperature, and pressure were 5.00 mol%, 281.15 K, and 3.00 MPa, respectively. The helium separation results at different *GLR* are quantitatively summarized in Table 2.

Table 2 shows that the *GSC*, which remains in the range of 76.21 Nm^3^/m^3^ to 73.12 Nm^3^/m^3^ from 140 Nm^3^/m^3^ *GLR* to 100 Nm^3^/m^3^ *GLR*, does not change significantly. In equilibrium gas, the helium concentration increases from 1.16 mol% at 140 Nm^3^/m^3^ to 1.66 mol% at 110 Nm^3^/m^3^, and the *RF* of helium remains almost unchanged at 96.00% to 97.88%. But when the *GLR* decreases from 110 Nm^3^/m^3^ to 100 Nm^3^/m^3^, the helium concentration increases from 1.66 mol% to 1.79 mol%, and the *RF* of helium significantly decreases from 96.00% to 89.10%. This is because when the *GLR* is small, as CH_4_ and C_2_H_6_ continue to be trapped in hydrate, the partial pressure of helium increases, resulting in the entrainment of helium into the hydrate phase, and the *RF* of helium significantly decreases.

In dissociated gas, as the *GLR* decreases from 140 Nm^3^/m^3^ to 110 Nm^3^/m^3^, the helium concentration does not change significantly. However, at the *GLR* of 100 Nm^3^/m^3^, the helium concentration is 0.08 mol%, which shows a significant increase compared to the *GLR* of 110 Nm^3^/m^3^, primarily due to the increase in helium molecules entrained in the hydrate. With the decrease in GLR, the CH_4_ concentration increases from 97.55 mol% to 98.35 mol%, which is almost unchanged. The C_2_H_6_ concentration in dissociated gas decreases from 2.43 mol% to 1.57 mol%. That indicates that as the *GLR* decreases, the proportion of CH_4_ in the hydrate cavities increases, while that of C_2_H_6_ decreases.

**Figure 4:**
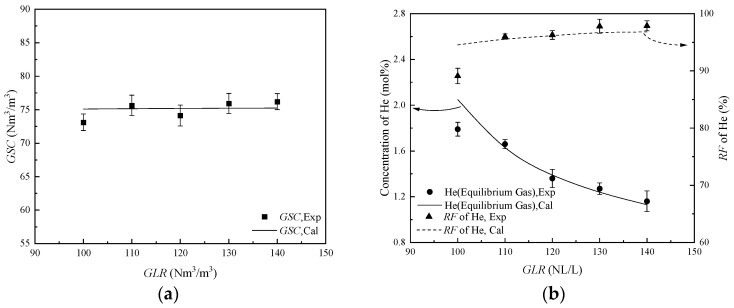
The helium separation performance from NG at different *GLR*. (**a**) The values of *GSC*; (**b**) The values of concentration of He and *RF* of He.

The comparison between the model-predicted and experimentally measured values of *GSC*, helium concentration, and *RF* of helium is shown in Figure 4. The *MRE* is 14.53% for a 100 Nm^3^/m^3^ *GLR* system, which is because, after the CH_4_ and C_2_H_6_ molecules are adsorbed into the hydrate cavities, the equilibrium gas volume is reduced, resulting in an increase in helium entrained into the hydrate slurry. That reduces the accuracy of the model calculations at low *GLR*. With an *ARE* of 2.62%, the mathematical model demonstrates a solid predictive accuracy for helium separation performance from NG at varying *GLR*. Considering the expectation of *GSC*, helium concentration, and *RF* of helium in industrial applications, 110 Nm^3^/m^3^ was used as the *GLR* for subsequent experiments.

#### 2.2.3. The Performance of HBGS at Different Temperatures

Since the THF solution needs to be cooled (hydrate formation) and heated (hydrate dissociation) in a hydrate formation–dissociation cycle, temperature governs the energy consumption of HBGS technology. The separation performances were studied from 279.15 K to 287.15 K, so that the selected temperature range not only meets the requirements of hydrate formation but also decreases the refrigeration load. The THF concentration, *GLR*, and pressure were 5.00 mol%, 110 Nm^3^/m^3^, and 3.00 MPa, respectively. The helium separation results at different temperatures are quantitatively summarized in Table 3.

As demonstrated in Table 3, *GSC* exhibits a negative correlation with temperature. That is attributed to the fact that decreased temperatures elevate hydrate molar yields, thereby boosting gas entrapment capacity in clathrate structures. Therefore, the *GSC* increases from 63.32 Nm^3^/m^3^ at 287.15 K to 75.65 Nm^3^/m^3^ at 281.15 K. The incremental gradient is about 2.06 (Nm^3^/m^3^)∙K^−1^. The amounts of CH_4_ and C_2_H_6_ trapped in the hydrate increase with the temperature decrease. In equilibrium gas, the helium concentration increases from 1.22 mol% at 287.15 K to 1.66 mol% at 281.15 K. The *RF* of helium, which is basically maintained at 95.87%~96.59%, is not significantly decreased. However, the *GSC* increases from 75.65 Nm^3^/m^3^ at 281.15 K to 77.15 Nm^3^/m^3^ at 279.15 K, and the incremental gradient is only about 0.75 (Nm^3^/m^3^)∙K^−1^. That indicates that the driving force was too high, and the hydrate shell effect appeared. With the increasing entrainment of helium molecules into the hydrate slurry, the helium concentration was limited to 1.64 mol%, and the *RF* of helium decreased to 90.70%.

In dissociated gas, the helium concentration remains stable within the temperature range of 287.15 K to 281.15 K. However, as the temperature decreases from 281.15 K to 279.15 K, the helium concentration increases from 0.03 mol% to 0.07 mol%. The CH_4_ concentration increases from 97.35 mol% to 98.55 mol%, and the C_2_H_6_ concentration decreases from 2.61 mol% to 1.38 mol% as the temperature decreases from 287.15 K to 279.15 K. It is obvious that the CH_4_ occupancy in the hydrate cavities increases, and the C_2_H_6_ occupancy decreases as the temperature decreases. This is because THF provides more hydrate cage structures to trap CH_4_ molecules at low temperature conditions, which enhances the CH_4_ proportion by the clathrate structure of hydrate.

**Figure 5:**
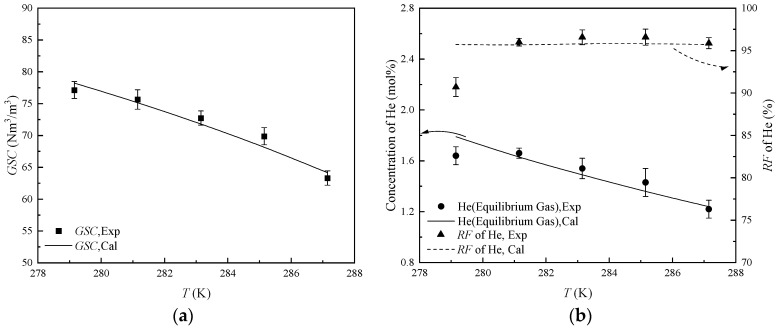
The helium separation performance from NG at different temperature. (**a**) The values of *GSC*; (**b**) The values of concentration of He and *RF* of He.

The comparison between the model-predicted and the experimentally measured values of *GSC*, helium concentration, and *RF* of helium is shown in Figure 5. The *MRE* of 9.15% appears in the 279.15 K system, which is still because, at low temperature, the trapping behavior of gas molecules by hydrate slurry includes not only the gas molecules captured by hydrate cavities but also the gas bubbles trapped by the hydrate. This results in the difference between the model-predicted and the experimentally measured values at low temperature. The *ARE* is 2.37%, indicating that the mathematical model accurately predicts the helium separation performance from NG at varying temperatures. Considering the expectation of *GSC*, helium concentration, and *RF* of helium in industrial applications, 281.15 K was used as the temperature for subsequent experiments.

#### 2.2.4. The Performance of HBGS Under Different Pressures

Pressure is the primary factor that determines the equipment cost of HBGS technology. The separation performance of helium extraction from helium-containing NG was investigated under a pressure range of 1.50 MPa to 3.50 MPa, so that the selected pressure range can meet the requirements of hydrate formation and not significantly increase the equipment cost. The THF concentration, *GLR*, and temperature were 5.00 mol%, 110 Nm^3^/m^3^, and 281.15 K, respectively. The helium separation results under different pressures are quantitatively summarized in Table 4.

As shown in Table 4, the *GSC* is positively correlated with the pressure. This is because an increase in pressure leads to more hydrate formation, which results in more capture of CH_4_ and C_2_H_6_. The *GSC* increases from 57.55 Nm^3^/m^3^ under 1.50 MPa to 75.65 Nm^3^/m^3^ under 3.00 MPa. The incremental gradient is approximately 12.07 Nm^3^/(m^3^∙MPa). Since the amount of CH_4_ and C_2_H_6_ captured in each mole of hydrate increases, the helium concentration in the equilibrium gas increases from 1.11 mol% under 1.50 MPa to 1.66 mol% under 3.00 MPa. Concurrently, the *RF* of helium experiences a decline from 98.01% to 96.00%. However, the *GSC* only increases from 75.65 Nm^3^/m^3^ under 3.00 MPa to 76.52 Nm^3^/m^3^ under 3.50 MPa, with an incremental gradient of only 1.74 Nm^3^/(m^3^∙MPa). That indicates the hydrate shell effect appears under higher driving forces, thereby constraining the conversion ratio of water into hydrate. As the amount of entrainment of helium molecules into the hydrate slurry increases, the helium concentration in the equilibrium gas is limited to 1.64 mol% under 3.5 MPa. And the *RF* of helium is limited to 92.44%.

In dissociated gas, the helium concentration remains relatively constant and has a minimal effect on the composition over the pressure range of 1.50 MPa to 3.00 MPa. But the helium concentration is 0.06 mol% under 3.50 MPa. The CH_4_ concentration increases from 97.55 mol% to 98.54 mol%, and the C_2_H_6_ concentration decreases from 2.43 mol% to 1.40 mol% as the pressure increases from 1.50 MPa to 3.50 MPa. The results indicate that the CH_4_ occupancy in the hydrate cavities increases, and the C_2_H_6_ occupancy in the hydrate cavities decreases. That is attributed to the fact that THF provides more hydrate cavity structures to trap CH_4_ molecules under high pressure, which increases the CH_4_ proportion by the clathrate structure of hydrate.

**Figure 6:**
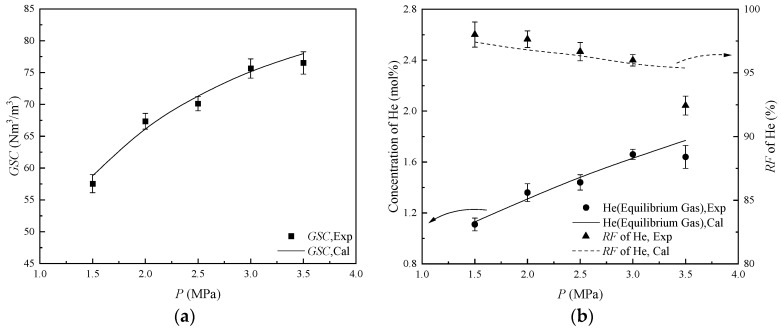
The helium separation performance from NG under different pressures. (**a**) The values of *GSC*; (**b**) The values of concentration of He and *RF* of He.

The comparison between the model-predicted and the experimentally measured values on the indexes of *GSC*, helium concentration, and *RF* of helium is shown in Figure 6. The *MRE* of 7.93% appears in a 3.50 MPa system, which is still because, under high pressure, the amount of helium entrained by the hydrate slurry increases. *ARE* is 2.11%, indicating that the mathematical model demonstrates an effective predictive accuracy for the helium separation performance from NG at varying pressure. Considering the expectation of *GSC*, helium concentration, and *RF* of helium in industrial applications, 3.00 MPa was used as the pressure for subsequent experiments.

### 2.3. The Performance of Three-Stage HBGS for the Helium Extraction from NG

**Figure 7:**
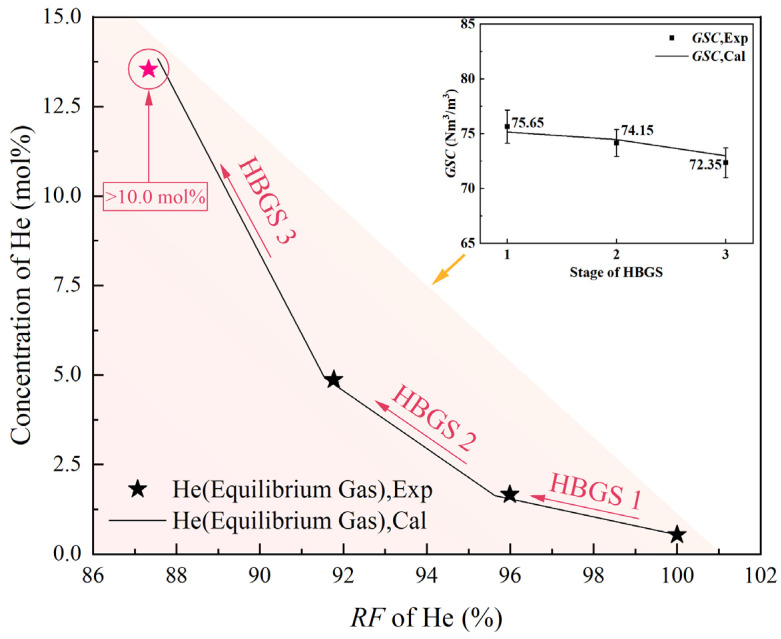
The separation process and the effect of the three-stage HBGS.

Above the performance of single-stage HBGS, the concentration of helium is enriched to 1.66 mol%. The three-stage HBGS process was investigated to further enrich the helium concentration because the helium concentrations exceeding 10.00 mol% in feed would substantially improve the energy efficiency of downstream purification technologies such as PSA [17]. At the optimized condition of 5.00 mol% THF solution, 110 Nm^3^/m^3^, 281.15 K, and 3.00 MPa, the experimental and calculated results detailing helium extraction efficiency across the three-stage HBGS are comprehensively presented in Table 5 and Figure 7.

Analysis reveals a distinct inverse correlation between the *GSC* and the separation stage number, with the *GSC* decreasing progressively from 75.65 Nm^3^/m^3^ at stage 1 to 72.35 Nm^3^/m^3^ at stage 3. Conversely, the helium concentration in the equilibrium gas phase exhibits a strong positive correlation with the stage number, increasing from the initial feed concentration of 0.54 mol% to 13.54 mol% by stage 3. This represents a significant enrichment of 25.07-fold. The total *RF* of helium is 87.34%.

The final helium concentration of 13.54 mol% achieved after three-stage HBGS holds considerable practical significance. This level of enrichment makes the equilibrium gas stream highly suitable for efficient downstream PSA purification. It leverages the well-documented efficiency gains of PSA at elevated feed concentrations. This promises substantial reductions in specific energy consumption compared to processing dilute helium streams. The dissociated NG after each stage separation used as fuel combustion can improve the gas calorific value [37].

The robustness of the experimental data is confirmed by the excellent agreement between calculated model predictions and measured values, evidenced by an *ARE* of only 2.09%. This close alignment underscores the reliability of the presented results and validates the model’s applicability for simulating the multi-stage HBGS process under these conditions. These findings demonstrate the efficacy of multi-stage THF-promoted HBGS as a promising pre-concentration step for helium extraction, offering a pathway to significantly improve the overall energy efficiency and economic viability of helium recovery from NG sources.

## 3. Discussion

According to the experimental results in Section 2.1, the GLHE of the NG-deionized water system is much more sensitive to the change in temperature than that of the NG-THF solution system. Since hydrate formation is an exothermic process [38], a slight increase in temperature can cause the end of the hydrate formation of the NG-deionized water system, which causes the end HBGS, and that makes the HBGS of the NG-deionized water system hard to operate. In addition, NG cannot form hydrate without THF at a temperature above 283.15 under 5 MPa (according to the experimental results in Section 2.1), whereas the pressure in the NG transportation pipes in countries like China is normally around 5 MPa. This means the NG from the pipeline needs to be further compressed before it can go into the HBGS process of the NG-deionized water system, which causes high energy consumption. For the above reasons, the HBGS without a thermodynamic promoter (like THF) cannot be put into the application in industry.

The thermodynamic promoter should be used in HBGS, but a higher promoter concentration does not always lead to better performance. For the performance of THF on decreasing GLHE, the best THF concentration is 5.00 mol% rather than 6.00 mol%, because the promotion of THF on increasing the thermodynamic driving force cannot keep up with the increase in THF concentration, whereas the effect of THF on decreasing the activity of water keeps growing stronger as THF concentration increases [39]. Regarding the effect of THF on enhancing separation performance, the best THF concentration is 5.00 mol% rather than 5.50 mol%, because high THF leads to high thermodynamic driving force, and the “hydrate shell effect” is generated when the thermodynamic driving force is too strong [35,36]. This effect then limits the performance of HBGS, which has been discussed previously in earlier paragraphs in this work.

The NG processing capacity of the THF solution system is limited by *GSC*, which is not higher than 80 Nm^3^/m^3^ in this work. Since the thermodynamic driving force primarily comes from THF, the change in the composition of the gas phase cannot cause a significant change in thermodynamic driving force, so *GLR* has no significant effect on *GSC*. Except for increasing THF concentration, the thermodynamic driving force can also be increased by decreasing temperature or increasing pressure, but the “hydrate shell effect” is generated when pressure is higher than a specific value (or temperature is lower than a specific value). For that reason, higher pressure and lower temperature do not always lead to better performance; there is an optimal temperature–pressure for the HBGS of NG, which is 281.15 K-3.00 MPa in this work.

Three-stage HGBS meets the demand for crude helium gas production from NG, but the performance on helium purification grows slightly weaker as the number of separation stages increases (helium concentration increases 2.1 times after stage 1, increases 1.9 times after stage 2, and increases 1.8 times after stage 3). HBGS purifies helium by capturing the other components in NG; the more helium in the feed gas, the fewer other components for the hydrate to capture.

The mathematical model can accurately predict the performance of HBGS with 2.09% average relative error compared to the experimental data, and that error is more significant under the operation conditions that have “hydrate shell effect”. That may be because the state of the end of the HBGS in the prediction model is near the thermodynamic equilibrium state, whereas that at the end of the HBGS that shows “hydrate shell effect” is farther away from the thermodynamic equilibrium state. The HBGS that shows “hydrate shell effect” is not economical, which has been discussed previously in this work, and the mathematical model in this work meets the requirements of industrial application. Based on the energy balance analysis of the pilot-scale facility at the Maoming Branch of Sinopec Corporation, the energy consumption of the three-stage gas separation process in this work is estimated to be less than 0.1 kWh/Nm^3^. As the length of a paper is limited and this paper focuses on separation performance and modeling, the energy consumption of the hydrate-based separation of helium-containing natural gas was not investigated in this work and will be systematically investigated in our future work.

## 4. Materials and Methods

### 4.1. Materials and Apparatus

A simulated helium-containing NG mixture (97.89 mol% CH_4_, 1.65 mol% C_2_H_6_, 0.54 mol% He), supplied by Karamay Zhongke Gas Co., Ltd. (Karamay, China), served as the feed gas. Gas composition analysis was performed using an Agilent 8890 gas chromatograph (Agilent Technologies, Santa Clara, CA, USA). Tetrahydrofuran (THF, purity ≥ 99.9%, Shanghai Aladdin Biochemical Technology Co., Ltd. (Shanghai, China)) was employed as the hydrate promoter. Aqueous THF solutions were prepared with deionized water (18.0 MΩ cm resistivity).

**Figure 8:**
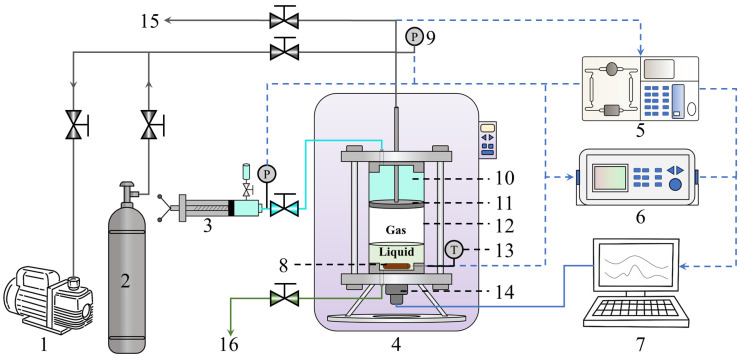
Apparatus for gas separation experiments. 1. Vacuum pump; 2. Gas cylinder; 3. Hand pump; 4. Air bath; 5. Gas chromatography; 6. Data acquisition; 7. Computer; 8. Magnetic stirrer; 9. Pressure transmitter; 10. Glycol solution; 11. Piston; 12. Equilibrium cell; 13. Platinum resistor; 14. Servo motor; 15. Gas outlet; 16. Liquid inlet/outlet.

Figure 8 illustrates the experimental apparatus, featuring a high-pressure equilibrium cell (maximum volume: 465.00 mL; maximum resistant pressure: 30.00 MPa) as the core component for determining GLHE conditions and conducting gas separation. System pressure, regulated via a hand pump acting on a movable piston, was monitored using a SnexDG2111 pressure transmitter (range: 20.00 MPa, accuracy: 0.1% full scale, Senex, Guangzhou, China). Precise temperature control within the range of 253.15 K to 343.15 K was achieved using an air bath, with temperature measured by an Omega Pt 100 probe (±0.01 K accuracy, Omega, New York, NY, USA). A computer-coupled data acquisition system recorded temperature, pressure, and processed gas composition data. The experimental gases used in this work are all pre-mixed gases. During the experiment, a magnetic stirrer was used to continuously stir the liquid phase, thereby enhancing the gas–liquid mass transfer. Since the experimental gas was under high pressure and the equilibrium cell is a batch reactor, the gas phase was uniform [40], so that only liquid was stirred during the experiments, and that stirring method is commonly employed in the hydrogen separation process using the hydrate method [39,41]. Since THF is corrosive, all seals in the experimental setup are made of corrosion-resistant rubber. Leak tests were conducted before and after each experiment to ensure the reliability of the experiments. The experimental setup was also used in our previous work, and its reliability has been verified [18,39].

### 4.2. Experimental Procedure

Measurement of GLHE conditions: The GLHE conditions by the pressure search method have been validated in previous work [41,42]. After washing the equilibrium cell, 50.00 mL of a prepared THF solution was injected into the cell. Then the air in the apparatus was evacuated, and the simulated NG was injected. Once the temperature in the cell was lowered to the desired temperature, the gas pressure was rapidly increased, leading to the formation of the hydrate. And then the gas pressure was subsequently decreased quickly, causing the hydrate to dissociate completely. To eliminate the hysteresis effect, the procedures of hydrate formation and dissolution were repeated twice. After that, the gas pressure was then gradually increased at a rate of 0.02 MPa/10 min until hydrate particles were observed in the cell, and then the gas pressure was stopped increasing. If the gas pressure remains stable for four hours, the pressure at that point in time is considered the GLHE pressure. The measurement was repeated three times, and the average of the three results was recorded.

Measurement of gas separation: The gas separation procedure of the HBGS method has been described in detail in previous studies [18,39]. The procedure initiates with triple rinsing of the equilibrium cell with the prepared THF solution. Then the cell volume is adjusted to meet experimental requirements via the hand pump. The THF solution with 50 mL is injected into the cell. The apparatus is then evacuated using a vacuum pump after purging residual air with the helium-containing NG. Then, the cell is cooled via an air bath to the target experimental temperature. The required volume of helium-containing NG is charged into the cell under constant magnetic stirring at 600 rpm, marking the start of the separation experiment. During hydrate formation, continuous feed gas consumption necessitates manually maintaining the target pressure by adjusting the hand pump. Equilibrium is attained when the system pressure remains constant for 4 h. After equilibrium is reached, the equilibrium gas phase (rich helium) is sampled for compositional analysis. The residual gas is then rapidly vented, and the cell temperature is increased to 303.15 K to dissociate the hydrate phase. The dissociated gas (poor helium) is collected for compositional analysis. To ensure reproducibility and statistical significance, all experimental conditions were repeated three times.

Experimental data processing: For each gas separation trial, the final gas absorption ($n_{diss}$) by hydrate derives from the calculated approach detailed as Equation (2).

(2) $$n_{diss}=\frac{P_{exp}\cdot {∆V}_{pump}}{Z_{exp}\cdot R\cdot T_{exp}}$$

where ${∆V}_{pump}$ is the volume change in the hand pump during the experiment. R is the gas constant. $P_{exp}$ and $T_{exp}$ are the pressure and temperature at the experimental condition, respectively. $Z_{exp}$ is the compression factor, which is calculated by the Patel–Teja equation of state (P-T EOS) [43]. It needs to be noted that the properties of helium are significantly different from those of methane, ethane, and other gases; errors may be present in the compression factors calculated using the equation of state. Since that kind of problem is also in the separation of the gas, including hydrogen, and helium’s properties are like hydrogen’s, we processed the data according to the method for the separation of the gas, including hydrogen, in the published literature [40,44]. In addition, the helium content in the gas in this work is much lower than the hydrogen content in the gas in the published studies [40,44]. The errors that came from helium are much smaller than those that came from hydrogen in the published literature [40,44]. To characterize the gas processing capacity, *GSC* is calculated using Equation (3).

(3) $$GSC=\frac{n_{diss}\cdot Z_{sc}\cdot R\cdot T_{sc}}{P_{sc}\cdot V_{liq}}$$

where $P_{sc}$, $T_{sc},$ and $Z_{sc}$ are the pressure, temperature, and compression factor at standard conditions, respectively. $V_{liq}$ is the volume of liquid. *RF* is calculated by Equations (4)–(6).

(4) $$RF=\left(1-\frac{(n_{feed}-n_{diss}){\cdot y}_{He}}{n_{NG}\cdot z_{He}}\right)\times 100\%$$

(5) $$n_{feed}=\frac{P_{exp}\cdot V_{feed}}{Z_{exp}\cdot R\cdot T_{exp}}$$

(6) $$V_{feed}=(V_{liq}\cdot GLR)\cdot \frac{P_{sc}}{P_{exp}}\cdot \frac{T_{exp}}{T_{sc}}\cdot \frac{Z_{exp}}{Z_{sc}}$$

where $V_{feed}$ and $n_{feed}$ are the volume and amount of feed gas injected into the equilibrium cell according to experimental conditions, respectively. *GLR* is the gas–liquid rate. $y_{He}$ and $z_{He}$ are the molar fractions of helium in the equilibrium gas and feed gas, respectively.

### 4.3. Mathematical Model

A novel theoretical model was developed to predict the performance of HBGS for three-component mixtures (CH_4_, C_2_H_6_, He). This model enables systematic evaluation of the THF concentration, gas–liquid rate, temperature, and pressure on the gas separation. The model employs a differential approach to characterize the hydrate formation process, discretizing the reaction timeline into sufficiently small increments. Throughout discrete time increments, compositional fluctuations across the gas–liquid–hydrate multiphase system exhibit negligible magnitudes. This fundamental assumption permits the application of phase equilibrium thermodynamics at each discrete interval.

Prior to hydrate formation, the equilibrium cell contains two distinct phases: the simulated NG and the THF solution.

(7) $$y_{i}^{t=0}=z_{i}$$

(8) $$n_{{gas}_{i}}^{t=0}=n_{feed}\cdot y_{i}^{t=0}$$

(9) $$n_{{hyd}_{i}}^{t=0}=0$$

(10) $$n_{THF}^{t=0}=m_{THF}^{t=0}\cdot n_{liq}$$

(11) $$n_{H_{2}O}^{t=0}=(1-m_{THF}^{t=0})\cdot n_{liq}$$

where z and y represent the molar fraction of components in the feed gas and the equilibrium gas, respectively. The subscript “*i*” represents the type of gas. i=I,II,III. “I” represents methane, “II” represents ethane, and “III” represents helium. The superscript t = 0 represents the time of hydrate formation. $n_{gas}$, $n_{feed}$, $n_{hyd}$ represent the molar amounts of different types of gases in the residual gas, feed gas, and hydrate phase, respectively. $n_{H_{2}O}$ and $n_{liq}$ represent the molar amounts of water and liquid, respectively. $m_{THF}$ is the molar fraction of THF in the liquid. As hydrates selectively capture different gas molecules, the composition in different phases changes. The driving force for hydrate formation continues to decrease. The driving force is described by chemical potential difference (−∆μ) [40]:

(12) $$∆\mu =RT\left[\lambda_{2}\ln\frac{f_{THF}^{t=0}}{f_{THF}}+\lambda_{1}\ln\left(1-\sum_{i=I}^{III}\theta_{i}\right)\right]$$

(13) $$\theta_{i}=\frac{f_{i}\cdot C_{i}}{1+\sum_{i=I}^{III}\left(f_{i}\cdot C_{i}\right)}$$

where $\lambda_{1}$ and $\lambda_{2}$ represent the number of small and large cavities contained in each water molecule within a hydrate crystal unit, respectively. $\theta_{i}$ and $C_{i}$ are the occupancy rate of gas molecules in the small cavities and the Langmuir adsorption constants, respectively. $f_{i}$ is the gas phase fugacity of gas. $f_{THF}$ and $f_{THF}^{0}$ are the fugacity of liquid and empty hydrate, respectively. The reaction rate ($r^{j}$) and the molar amount of THF consumed ($∆n_{THF}^{j}$) at the *j*th time element are described as Equations (14) and (15) [39].

The meaning of k, as well as the method to determine the value of k, has been discussed and investigated systematically in our previous work [39]. *k* is the hydrate-forming rate parameter, and it is affected by the kinetic influential factors such as temperature, pressure, interphase surface area, and so on, theoretically. However, the prediction model in this work is used to calculate the separation results at the end of separation (the system reaches thermodynamic equilibrium) rather than to calculate the separation results during the separation process. For that reason, k can be set at a fixed value, which is not affected by any influential factors. The smaller k is, the smaller the difference between the calculated results at the *j*th time element and the calculated results at the (*j* − 1)th time element. For that reason, for the calculation with a specific uncertainty, the decrease in k would not lead to any change in the final calculated results when *k* is lower than a specific value. That means that, when k is lower than the specific value, *k* can only affect the number of iterations of the calculation but cannot affect the calculation results. In this work, the uncertainties of the calculation are specific and fixed (the uncertainties of calculated gas composition are ±0.005 mol%, the uncertainties of calculated *GSC* are ±0.005 Nm^3^/m^3^, the uncertainties of calculated *RF* are ±0.005%), according to the method to determine the value of k in our previous work [39], *k* was set at 0.005 mol water^−1^·Δt^−1^, and the decrease in *k* would not lead to any change in the final calculated results when *k* was lower than 0.005 mol water^−1^·Δt^−1^ [39].

(14) $$r^{j}=k\cdot n_{H_{2}O}^{j-1}\cdot \left(exp\left(\frac{-∆\mu_{j-1}}{RT}\right)-1\right)$$

(15) $$∆n_{THF}^{j}=r^{j}\cdot t$$

where *k* represents the constant of hydrate formation. The mole amount of different gas molecules trapped in the hydrate at the *i*th time element ($∆n_{{hyd}_{i}}^{j}$) is calculated as follows:

(16) $$∆n_{{hyd}_{i}}^{j}=\frac{\lambda_{1}}{\lambda_{2}}\cdot \theta_{i}^{j}\cdot ∆n_{THF}^{j}$$

The chemical potential difference (−∆μ) governs hydrate behavior, including hydrate formation (−∆μ > 0), hydrate dissociation (−∆μ < 0), and the termination of separation iteration (−∆μ ≈ 0). The molar amounts of CH_4_, C_2_H_6_ and He in the gas phase ($n_{{gas}_{i}}^{j}$) and hydrate phase ($n_{{hyd}_{i}}^{j}$) at the *j*th time element are calculated by Equations (17) and (18).

(17) $$n_{{gas}_{i}}^{j}=n_{{gas}_{i}}^{j-1}-∆n_{{hyd}_{i}}^{j}$$

(18) $$n_{{hyd}_{i}}^{j}=n_{{hyd}_{i}}^{j-1}+∆n_{{hyd}_{i}}^{j}$$

The mole amount of consumed THF and H_2_O during the *j*th temporal interval is described as Equations (19) and (20).

(19) $$n_{THF}^{j}=n_{THF}^{j-1}-∆n_{THF}^{j}$$

(20) $$n_{H_{2}O}^{j}=n_{H_{2}O}^{j-1}-\frac{1}{\lambda_{2}}∆n_{THF}^{j}$$

The phase-specific compositions ($y_{i}^{j}$ for gas, $x_{i}^{j}$ for hydrate) during the *j*th temporal interval are determined by Equations (21) and (22).

(21) $$y_{i}^{j}=\frac{n_{{gas}_{i}}^{j}}{\sum_{j=I}^{III}\left(n_{{gas}_{i}}^{j}\right)}$$

(22) $$x_{i}^{j}=\frac{n_{{hyd}_{i}}^{j}}{\sum_{j=I}^{III}\left(n_{{hyd}_{i}}^{j}\right)}$$

**Figure 9:**
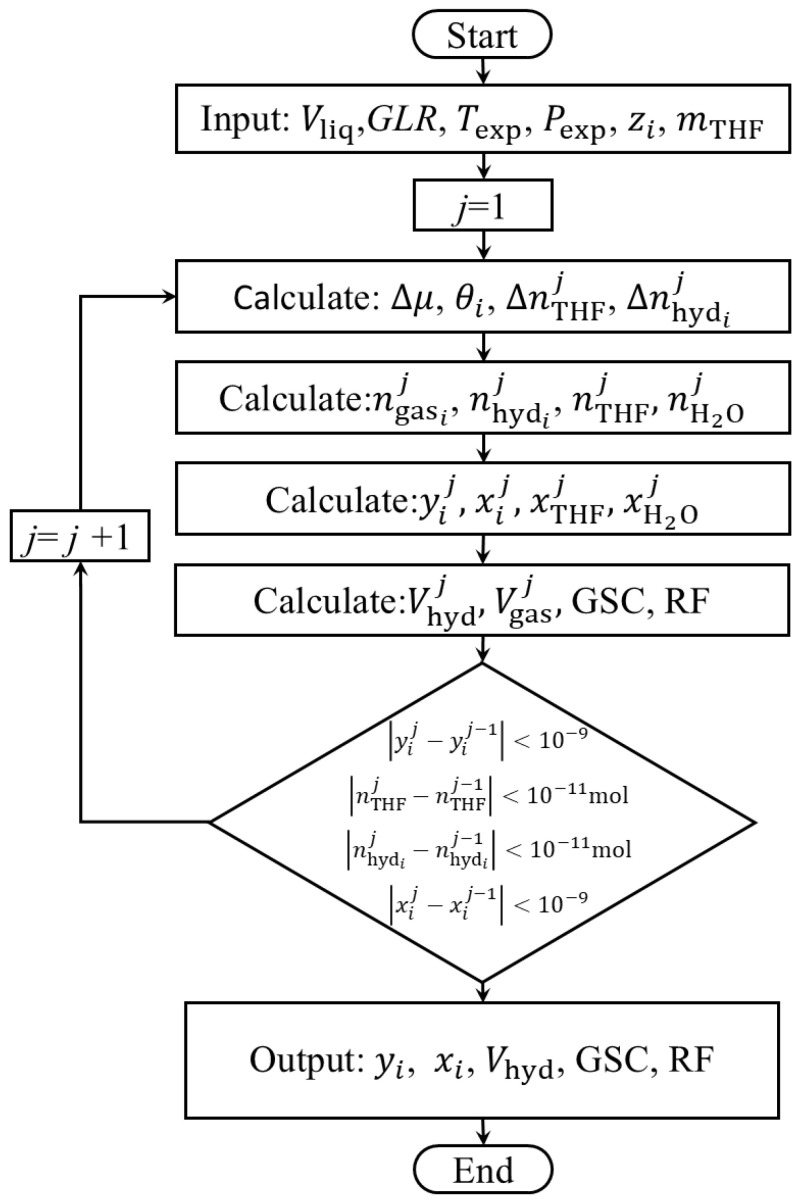
The separation process and the effect of the three-stage separation by HBGS.

The composition of substances in the liquid phase during the *j*th temporal interval is calculated by Equations (23)–(25).

(23) $$x_{THF}^{j}=\frac{n_{THF}^{j}}{n_{THF}^{j}+n_{H_{2}O}^{j}}$$

(24) $$V_{hyd}^{j}=\frac{(\sum_{j=I}^{III}\left(n_{{hyd}_{i}}^{j}\right))\cdot Z_{sc}\cdot R\cdot T_{SC}}{P_{sc}}$$

(25) $$V_{gas}^{j}=\frac{(\sum_{j=I}^{III}\left(n_{{gas}_{i}}^{j}\right))\cdot Z_{sc}\cdot R\cdot T_{SC}}{P_{sc}}$$

where $x_{THF}^{j}$ is the molar fraction of THF in the liquid phase. $V_{hyd}^{j}$ and $V_{gas}^{j}$ are the volumes of gas captured by the hydrate and the equilibrium gas, respectively. Figure 9 illustrates the calculation flowchart of HBGS.

## 5. Conclusions

Helium extracted from helium-containing NG (97.89 mol% CH_4_, 1.65 mol% C_2_H_6_, 0.54 mol% He) holds significant strategic importance due to the scarcity and the increasing global demand for helium. This study investigated a scheme for the crude separation of helium from NG by the HBGS method.

(1) Optimization of hydrate formation conditions: THF demonstrated a pronounced thermodynamic promotion effect. A 5.00 mol% THF solution was identified as optimal for minimizing the hydrate formation pressure of simulated NG. Within the temperature range of 279.15 K to 287.15 K, the GLHE pressure in a 5.00 mol% THF solution was decreased by 92.11% compared to a deionized water system.
(2) Single-stage separation performance: The measures of increasing THF concentration, decreasing temperature, and increasing pressure effectively increase the gas storage capacity and increase helium concentration in the equilibrium gas phase. However, these measures concurrently decrease the helium recovery factor. The measure of decreasing *GLR* increases helium concentration in the equilibrium gas but also decreases the recovery of helium while exhibiting no significant effect on the gas storage capacity. The maximum achievable helium concentration is limited by the hydrate shell effect.
(3) Multi-stage separation performance: A three-stage HBGS process successfully concentrates helium from 0.54 mol% in the feed to 13.54 mol% in the final equilibrium gas stream, representing a 25.07-fold increase, while maintaining a total helium *RF* of 87.34%.
(4) Predictive model development: A mathematical model is developed to predict HBGS performance for the helium extraction from NG. The calculated values show effective agreement with the experimental data, as evidenced by an absolute relative error of only 2.09%.

The results demonstrate the considerable potential of HBGS for efficiently separating helium from natural gas (NG). The developed predictive model is a valuable tool for future process design and optimization.

## Figures and Tables

**Table 1:** The helium separation results from NG at different THF concentrations.

Performance	The Concentration of THF				
	3.50 mol%	4.00 mol%	4.50 mol%	5.00 mol%	5.50 mol%
Equilibrium gas					
He (mol%)	1.04	1.19	1.42	1.66	1.65
CH_4_ (mol%)	98.22	97.93	97.51	97.16	96.82
C_2_H_6_ (mol%)	0.74	0.88	1.07	1.18	1.53
Dissociated gas					
He (mol%)	0.02	0.02	0.03	0.03	0.06
CH_4_ (mol%)	97.54	97.86	98.11	98.22	98.35
C_2_H_6_ (mol%)	2.44	2.12	1.86	1.75	1.59
Index					
*GSC* (Nm^3^/m^3^)	53.75	61.12	69.55	75.65	76.86
*RF* (He, %)	98.49	97.83	96.70	96.00	92.06

**Table 2:** The helium separation results from NG at different *GLR*.

Performance	*GLR*				
	100 Nm^3^/m^3^	110 Nm^3^/m^3^	120 Nm^3^/m^3^	130 Nm^3^/m^3^	140 Nm^3^/m^3^
Equilibrium gas					
He (mol%)	1.79	1.66	1.36	1.27	1.16
CH_4_ (mol%)	96.64	97.16	97.84	98.13	98.30
C_2_H_6_ (mol%)	1.57	1.18	0.80	0.60	0.54
Dissociated gas					
He (mol%)	0.08	0.03	0.03	0.02	0.02
CH_4_ (mol%)	98.35	98.22	97.92	97.72	97.55
C_2_H_6_ (mol%)	1.57	1.75	2.05	2.26	2.43
Index					
*GSC* (Nm^3^/m^3^)	73.12	75.65	74.13	75.94	76.21
*RF* (He, %)	89.10	96.00	96.27	97.80	97.88

**Table 3:** The helium separation results from NG at different temperatures.

Performance	Temperature				
	279.15 K	281.15 K	283.15 K	285.15 K	287.15 K
Equilibrium gas					
He (mol%)	1.64	1.66	1.54	1.43	1.22
CH_4_ (mol%)	96.34	97.16	97.58	98.31	98.62
C_2_H_6_ (mol%)	2.02	1.18	0.88	0.26	0.16
Dissociated gas					
He (mol%)	0.07	0.03	0.03	0.03	0.04
CH_4_ (mol%)	98.55	98.22	98.05	97.65	97.35
C_2_H_6_ (mol%)	1.38	1.75	1.92	2.32	2.61
Index					
*GSC* (Nm^3^/m^3^)	77.15	75.65	72.75	69.88	63.32
*RF* (He, %)	90.70	96.00	96.57	96.59	95.87

**Table 4:** The helium separation results from NG under different pressures.

Performance	Pressure				
	1.50 MPa	2.00 MPa	2.50 MPa	3.00 MPa	3.50 MPa
Equilibrium gas					
He (mol%)	1.11	1.36	1.44	1.66	1.64
CH_4_ (mol%)	98.26	98.11	97.49	97.16	96.40
C_2_H_6_ (mol%)	0.63	0.53	1.07	1.18	1.96
Dissociated gas					
He (mol%)	0.02	0.02	0.03	0.03	0.06
CH_4_ (mol%)	97.55	97.75	98.12	98.22	98.54
C_2_H_6_ (mol%)	2.43	2.23	1.85	1.75	1.40
Index					
*GSC* (Nm^3^/m^3^)	57.55	67.35	70.12	75.65	76.52
*RF* (He, %)	98.01	97.65	96.68	96.00	92.44

**Table 5:** The helium separation results from NG by three-stage HBGS.

Performance	Stage 1		Stage 2		Stage 3	
	Exp.	Cal.	Exp.	Cal.	Exp.	Cal.
Feed gas						
He (mol%)	0.54	0.54	1.66	1.66	4.87	4.87
CH_4_ (mol%)	97.89	97.89	97.16	97.16	94.33	94.33
C_2_H_6_ (mol%)	1.57	1.57	1.18	1.18	0.80	0.80
Equilibrium gas						
He (mol%)	1.66	1.63	4.87	4.92	13.54	13.84
CH_4_ (mol%)	97.16	97.44	94.33	97.41	85.39	85.73
C_2_H_6_ (mol%)	1.18	0.93	0.80	0.67	1.07	0.43
Dissociated gas						
He (mol%)	0.03	0.03	0.11	0.10	0.36	0.32
CH_4_ (mol%)	98.22	98.10	98.53	98.47	98.98	98.69
C_2_H_6_ (mol%)	1.75	1.87	1.36	1.43	0.66	0.99
Index						
*GSC* (Nm^3^/m^3^)	75.65	75.15	74.15	74.47	72.35	72.99
*RF* (He, %)	96.00	95.63	95.61	95.74	95.16	95.62

## Data Availability

The data that support the findings of this study are contained within the article. More information is available on request from the corresponding author.
